# Modeling absolute zone size in retinopathy of prematurity in relation to axial length

**DOI:** 10.1038/s41598-022-08680-5

**Published:** 2022-03-18

**Authors:** Sean K. Wang, Edward Korot, Moosa Zaidi, Marco H. Ji, Ahmad Al-Moujahed, Natalia F. Callaway, Jochen Kumm, Darius M. Moshfeghi

**Affiliations:** 1 Department of Ophthalmology, Byers Eye Institute, Horngren Family Vitreoretinal Center, Stanford University School of Medicine, 2452 Watson Court, Rm. 2277, Palo Alto, CA 94303 USA; 2 Department of Ophthalmology, Jones Eye Institute, University of Arkansas for Medical Sciences, Little Rock, AR USA

**Keywords:** Eye diseases, Retina, Physical examination, Retinopathy of prematurity

## Abstract

Treatment outcomes in retinopathy of prematurity (ROP) are closely correlated with the location (i.e. zone) of disease, with more posterior zones having poorer outcomes. The most posterior zone, Zone I, is defined as a circle centered on the optic nerve with radius twice the distance from nerve to fovea, or subtending an angle of 30 degrees. Because the eye enlarges and undergoes refractive changes during the period of ROP screening, the absolute area of Zone I according to these definitions may likewise change. It is possible that these differences may confound accurate assessment of risk in patients with ROP. In this study, we estimated the area of Zone I in relation to different ocular parameters to determine how variability in the size and refractive power of the eye may affect zoning. Using Gaussian optics, a model was constructed to calculate the absolute area of Zone I as a function of corneal power, anterior chamber depth, lens power, lens thickness, and axial length (AL), with Zone I defined as a circle with radius set by a 30-degree visual angle. Our model predicted Zone I area to be most sensitive to changes in AL; for example, an increase of AL from 14.20 to 16.58 mm at postmenstrual age 32 weeks was calculated to expand the area of Zone I by up to 72%. These findings motivate several hypotheses which upon future testing may help optimize treatment decisions for ROP.

## Introduction

Retinopathy of prematurity (ROP) is a disease seen in premature and low-birth-weight infants in which abnormal vascularization of the retina threatens vision. Although several treatment modalities for ROP, including laser, surgery, and anti-angiogenic agents, can help prevent its complications^1–3^, the condition remains a leading cause of childhood blindness and visual impairment^4–6^. To ensure timely treatment, babies at risk for or diagnosed with ROP are screened on a regular basis with the location of disease described by zones^7^. Disease in the most posterior zone, Zone I, is frequently associated with poor visual outcomes^8,9^, necessitating closer monitoring and intervention when this region is involved. Indeed, current guidelines from the American Academy of Pediatrics, American Academy of Ophthalmology, American Association for Pediatric Ophthalmology and Strabismus, and American Association of Certified Orthoptists recommend follow-up ≤ 1 week for any non-regressing Zone I ROP and urgent treatment in cases with higher severity (stage) or vascular changes (plus disease)^10,11^.

The location of Zone I in ROP was originally defined by the International Classification of ROP (ICROP) in 1984 as a circle centered on the optic nerve with radius twice the distance from nerve to fovea, or subtending 30 degrees^7^. Under this definition, eyes were classified according to the most posterior extent of retinal avascularity; even if one clock-hour of retina showed Zone I disease while the remainder were outside, the entire eye was classified as Zone I^7^. In 2005, the second ICROP (ICROP2) added another definition for Zone I that was practical but subjective: when using a 25- or 28-diopter lens with the optic nerve displaced to the nasal limit of the field of view, everything temporal falls within the confines of Zone I^12^. ICROP3 in 2021 largely retained these previous definitions for Zone I while introducing the “notch” terminology^13^. Eyes with more peripherally located disease with only a one to two clock-hour incursion of the ROP lesion into a more posterior zone are now classified as “with a notch” to distinguish them from those with more posterior disease.

**Figure 1:**
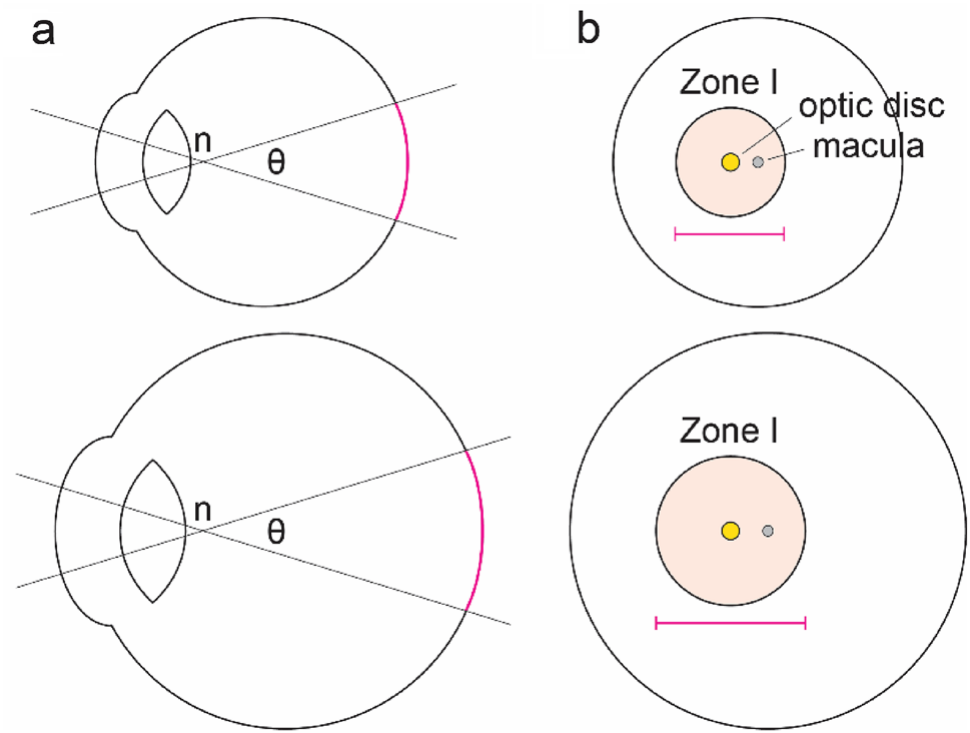
Neonatal eye development in ROP may affect zoning. (**a**) Schematic of a growing eye with the nodal point and nodal angle denoted by n and θ, respectively. As the eye becomes larger and undergoes refractive changes, the area of the region (magenta) encompassed by θ may likewise change. (**b**) Schematic of the retina in a growing eye. Although both regions in beige are defined as Zone I, the absolute areas corresponding to Zone I might differ substantially.

While these above definitions have simplified the diagnosis of Zone I ROP during ophthalmic exams, they do not account for the dynamic nature of the newborn eye. In reality, the eye undergoes substantial postnatal growth^14–21^, meaning that the absolute area of Zone I as encompassed by a 30-degree visual angle will change over time (Fig. 1a,b). Other ocular parameters such as corneal and lens power also change during the period of ROP screening^14,19,22^, causing variations that might affect how Zone I is viewed.

Additionally, there is evidence that the absolute amount of avascular retina in ROP negatively correlates with treatment responses. Indeed, Zone I eyes, which have the largest area of avascular retina, have the worst outcomes, while the reverse is true for eyes with Zone III ROP^9^. Among treatment options, cryotherapy typically addresses a smaller area of avascular retina than laser photocoagulation due to the physical limitations of the cryotherapy probe. Cryotherapy likewise has been found to underperform laser photocoagulation^23^, suggesting that the amount of untreated retina matters. Within laser photocoagulation, outcomes are improved with denser laser patterns or fewer skip lesions^24–26^, both of which result in less remaining avascular retina. Finally, Chen et al. found a correlation between the area of ischemia and ROP reactivation in eyes receiving intravitreal bevacizumab^27^. Consistent with this, animal models of ROP have shown a strong association between the total area of avascular retina and the incidence of neovascularization^28,29^.

Given the rising incidence of ROP and the potentially devastating consequences of delayed care^30^, it is critical that our definition of what constitutes treatment-warranted disease is precise and reproducible for each patient and eye. Furthermore, with the emergence of machine-learning based algorithms for detecting ROP^31,32^, explicitly defining ground truth parameters becomes increasingly important. The purpose of this study was to estimate the absolute area of ICROP Zone I during neonatal development to determine how variability in the length and refractive power of the eye affects zoning. To calculate Zone I area, we used a schematic model of the eye and incorporated data on corneal power (P_cornea_), anterior chamber depth (ACD), lens power (P_lens_), lens thickness (LT), and axial length (AL). Our model predicts that the area of Zone I as currently defined by the ICROP could grow considerably during ROP screening with increasing AL. These findings motivate several hypotheses which upon future testing may help optimize treatment decisions for ROP.

## Methods

A model was developed to estimate the absolute area of Zone I by representing it as a circle with radius set by a 30-degree visual angle from the posterior nodal point (Supplementary Eqs. 1 and 2). Input variables for this model were P_cornea_ (in diopters), ACD (in mm), P_lens_ (in diopters), LT (in mm), and AL (in mm). First, the position of the nodal point was calculated using methods described by Snead et al.^33^, which employ the refractive powers of the cornea and lens and the distance between them to determine the nodal point location. These calculations assumed a refractive index of 1.3375 for the cornea and aqueous humor, 1.4000 for the lens and vitreous humor, and 1.0000 for air, as well as a symmetric thin lens located ACD + (LT/2) from the cornea. The position of the posterior nodal point relative to the cornea was then subtracted from AL to determine the distance from the nodal point to the back of the eye. This value was multiplied by tan(30°) to obtain the radius of Zone I, which was squared and multiplied by pi to estimate the area of the Zone I circle.

Values for P_cornea_, ACD, P_lens_, LT, and AL at specific postmenstrual ages (PMAs) were estimated using published datasets (Table 1)^14–22^. For each dataset, the relationships between ocular parameters and PMA were represented as equations (Supplementary Table 1). The given week was inputted into these equations to generate a list of possible values for that ocular parameter. For studies in which the authors mathematically described how their parameters related to PMA, the equations presented in the original study were used. For all other studies, published data on ocular parameters were fitted with a second-order polynomial equation using Microsoft Excel (Microsoft, Redmond, WA) with an average R^2^ value of 0.915. If data for corneal curvature rather than P_cornea_ were reported, P_cornea_ was calculated as 337.5 divided by the radius of curvature. In one study in which refractive error data was additionally available, P_lens_ was obtained using the Hoffer Q formula^34^.

**Table 1:** List of studies measuring ocular parameters in preterm infants.

Parameter	Study	# eyes
Corneal power (P_cornea_)	Gordon and Donzis, 1985	35
	Inagaki, 1986	16
	Cook et al., 2003	27
Anterior chamber depth (ACD)	Isenberg et al., 1995	101
	Cook et al., 2003	38
	Ozdemir et al., 2015	361
Lens power (P_lens_)	Gordon and Donzis, 1985	35
	Cook et al., 2003	27
Lens thickness (LT)	Cook et al., 2003	38
	Ozdemir et al., 2015	361
Axial length (AL)	Gordon and Donzis, 1985	35
	Tucker et al., 1992	140
	O'Brien and Clark, 1994	473
	Isenberg et al., 1995	101
	Fledelius and Christensen, 1996	101
	Cook et al., 2003	38
	Ozdemir et al., 2015	361
	Kardaras et al., 2019	200

Ranges for Zone I area were generated by entering each possible combination of ocular parameter values into the model, from which the smallest and largest possible outputs were identified. Data were analyzed using Microsoft Excel and visualized using GraphPad Prism (GraphPad, San Diego, CA). Schematics and figures were assembled using Adobe Illustrator (Adobe, San Jose, CA).

All clinical measurements used in this study were obtained from publicly available datasets and deidentified. Research was conducted with institutional review board approval from the Stanford University School of Medicine and adhered to the tenets of the Declaration of Helsinki.

## Results

**Figure 2:**
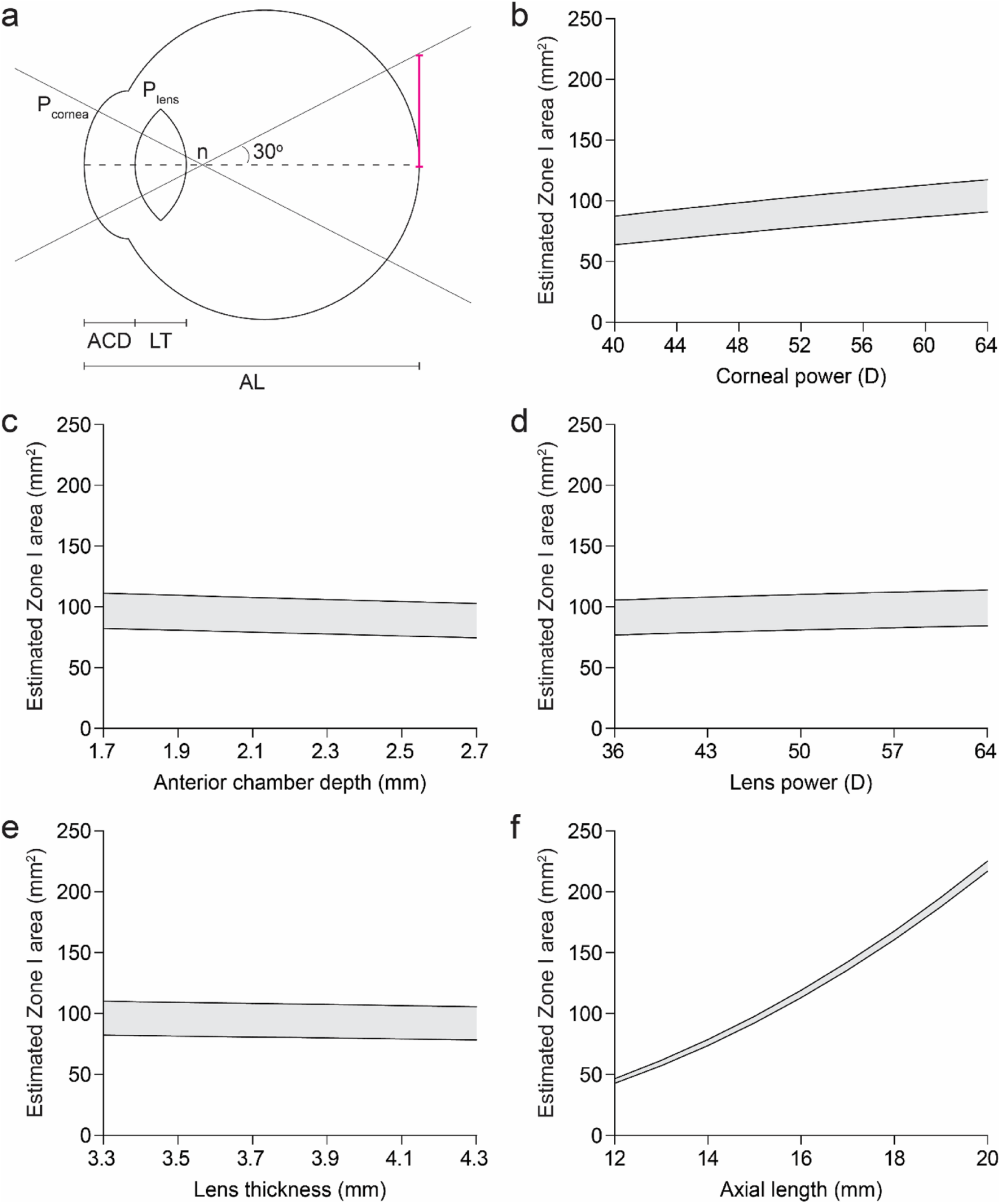
Zone I area is most sensitive to changes in axial length. (**a**) Ocular parameters used to estimate the absolute area of ICROP Zone I. After determining the position of the nodal point (n) based on P_cornea_, P_lens_, ACD, and LT, the radius (magenta) of the circle corresponding to Zone I was calculated as tan(30°) times the distance from the nodal point to the back of the eye. For further details, see Methods. (**b**–**f**) Range of estimated Zone I areas at PMA 31 weeks when modifying only corneal power (b), anterior chamber depth (**c**), lens power (**d**), lens thickness (**e**), or axial length (**f**).

To estimate the effect of variation in ocular parameters on the area of ICROP Zone I, we employed a schematic model of the eye involving P_cornea_, ACD, P_lens_, LT, and AL (Fig. 2a). Zone I in this model was represented as a circle at the back of the eye with radius set by a 30-degree visual angle emanating from the nodal point. We first examined how isolated adjustments to each ocular parameter in our model affected the calculated area of Zone I. When P_cornea_, ACD, P_lens_, and LT were each modified alone with all other parameters held constant, the resulting changes in Zone I area were relatively modest (Fig. 2b–e). Conversely, we found that fluctuations in AL had large effects on the estimated area of Zone I (Fig. 2f).

**Figure 3:**
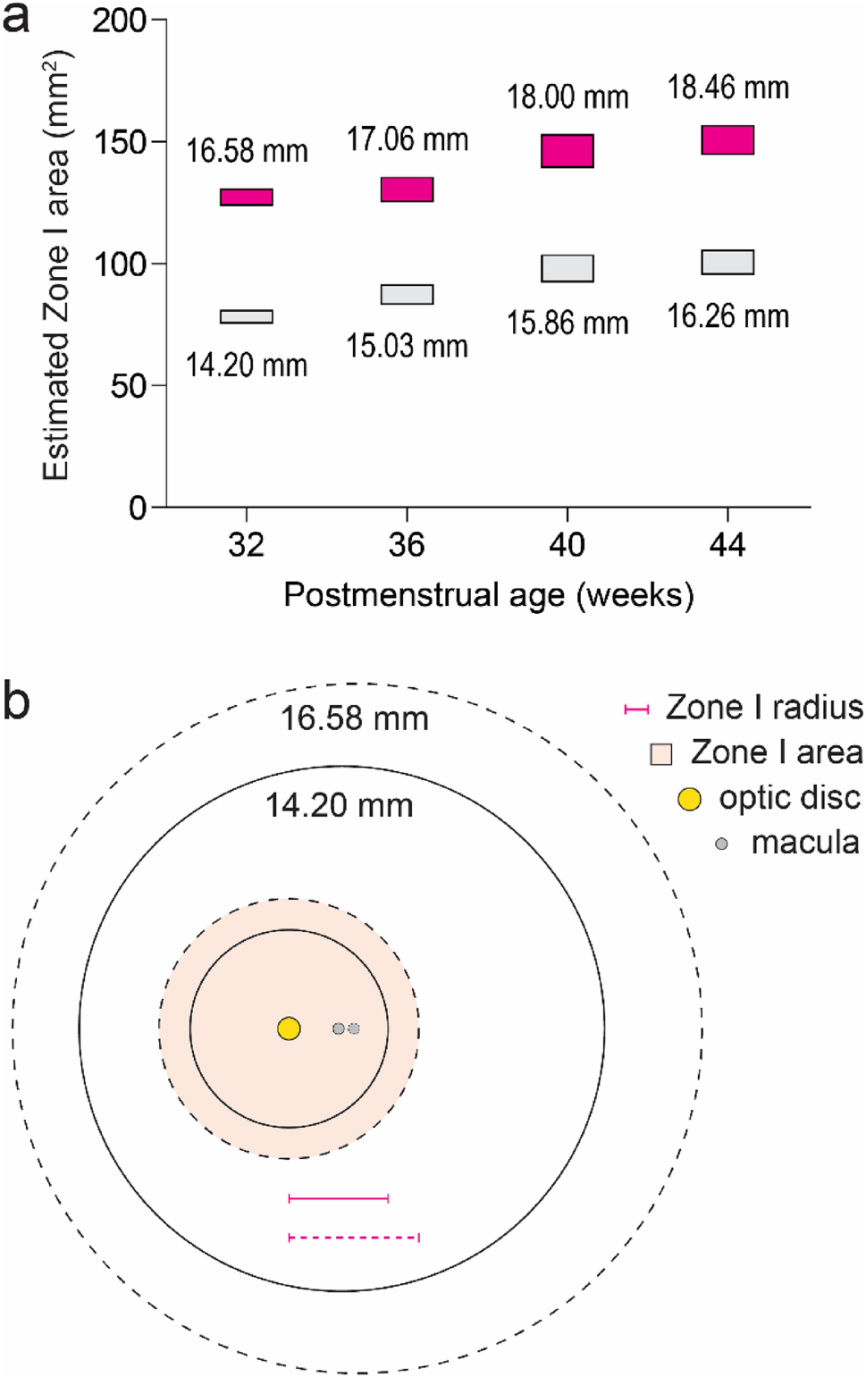
Zone I area increases in relation to axial length. (**a**) Range of estimated Zone I areas at indicated ages when using the lowest (gray) and highest (magenta) AL values reported by Cook et al.^19^. (**b**) Depiction of a 72% increase in retina and Zone I areas at PMA 32 weeks based on model predictions following a change in AL from 14.20 (solid lines) to 16.58 (dashed lines) mm.

We next searched published datasets for AL measurements from individual subjects and identified one study in which minimum and maximum values at specific ages were reported^19^. Using the lowest and highest ALs observed at each age in this cohort, we calculated the area of ICROP Zone I in our model. At PMA 32 weeks, Zone I in the longest eye (AL = 16.58 mm) was predicted to be up to 72% larger than in the shortest eye (AL = 14.20 mm) (Fig. 3a,b). Analyses using AL values from other ages revealed similar changes, with predicted area differences of up to 62%, 65%, and 64% calculated at PMA 36, 40, and 44 weeks, respectively.

**Figure 4:**
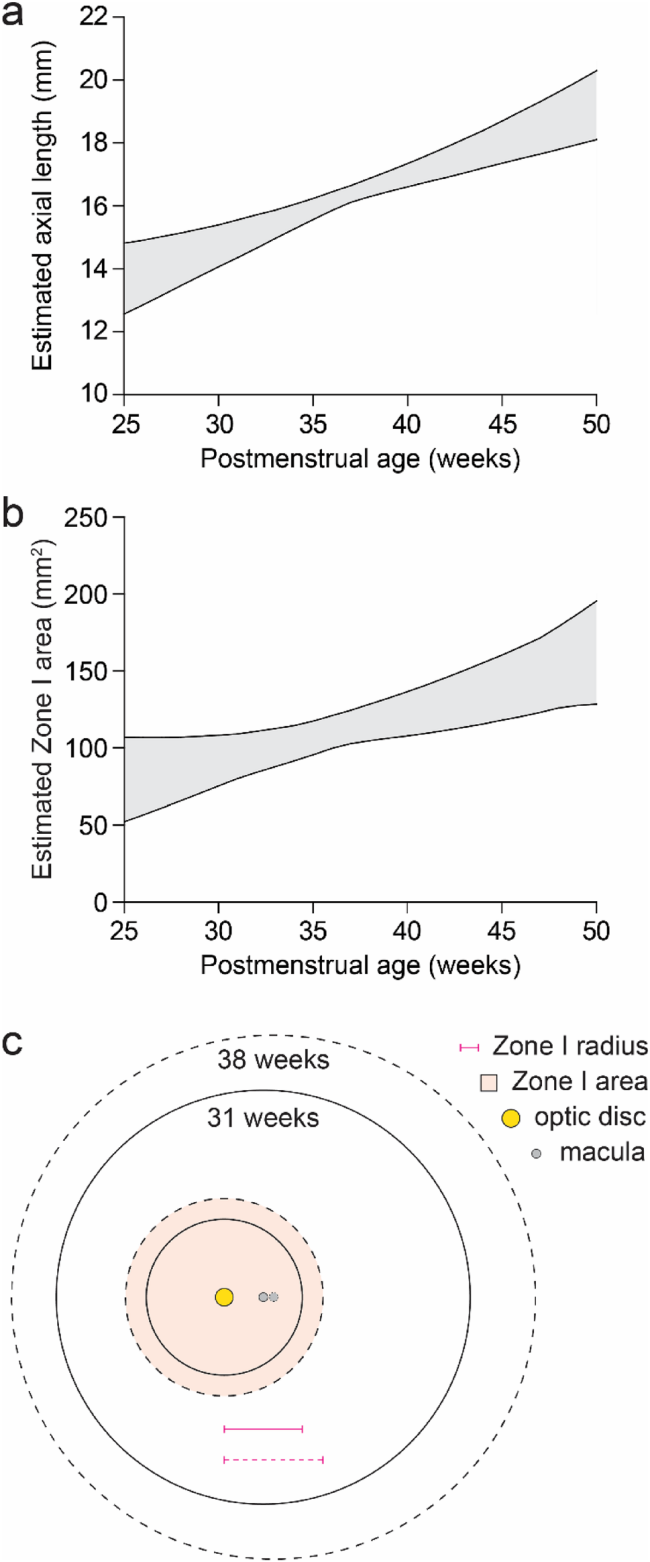
Postmenstrual age can approximate axial length to model zone area. (**a**) Range of estimated AL values at different PMAs. (**b**) Range of estimated ICROP Zone I areas at different PMAs. (**c**) Depiction of a 60% increase in retina and Zone I areas from PMA 31 (solid lines) to 38 (dashed lines) weeks based on model predictions.

Because PMA correlates with AL (Fig. 4a), we then calculated the area of Zone I in relation to PMA to model cases in which AL measurements are not available. In the youngest eyes at PMA 25 weeks, we estimated the area of Zone I to be as small as 52 mm^2^ (Fig. 4b). In contrast, the area of Zone I at PMA 50 weeks was calculated to be as large as 196 mm^2^, nearly four-fold greater. From PMA 31 weeks, when many premature babies are first screened^11,35^, to 38 weeks, a common age for treatment^36^, our model predicted that the absolute area of ICROP Zone I could expand by up to 60% (Fig. 4c). Our model thus predicts that retinal zones as currently defined by the ICROP could grow considerably during ROP screening due to increasing AL.

## Discussion

Current recommendations for treatment and follow-up in ROP consider zone, stage, and plus disease^11,37^, but not other potential contributing factors such as the size of the eye and retina at exam. Nonetheless, because eye size and refraction during the postnatal period are in flux, we hypothesized that these parameters could influence retinal zoning. Here, we constructed a model to calculate the absolute area of ICROP retinal zones, which predicted zone areas to change during ROP screening particularly with AL. These findings generate several hypotheses regarding the classification and management of ROP which are discussed below.

First, because the area of ICROP Zone I should be smaller in shorter eyes, it is possible that Zone I ROP eyes with shorter ALs may have worse outcomes than those with longer ALs. For instance, although eyes with Zone I ROP and AL of 13 mm versus Zone I ROP and AL of 17 mm are currently equivalently managed, the disease may be closer to the macula in the former, where it might pose a higher threat to vision. While we are not aware of any datasets which directly support or refute this hypothesis, this question could potentially be answered by a prospective study of Zone I ROP patients in which ALs are measured. These measurements could be obtained at the time of ROP screening using widely available methods such as applanation A-scan.

**Figure 5:**
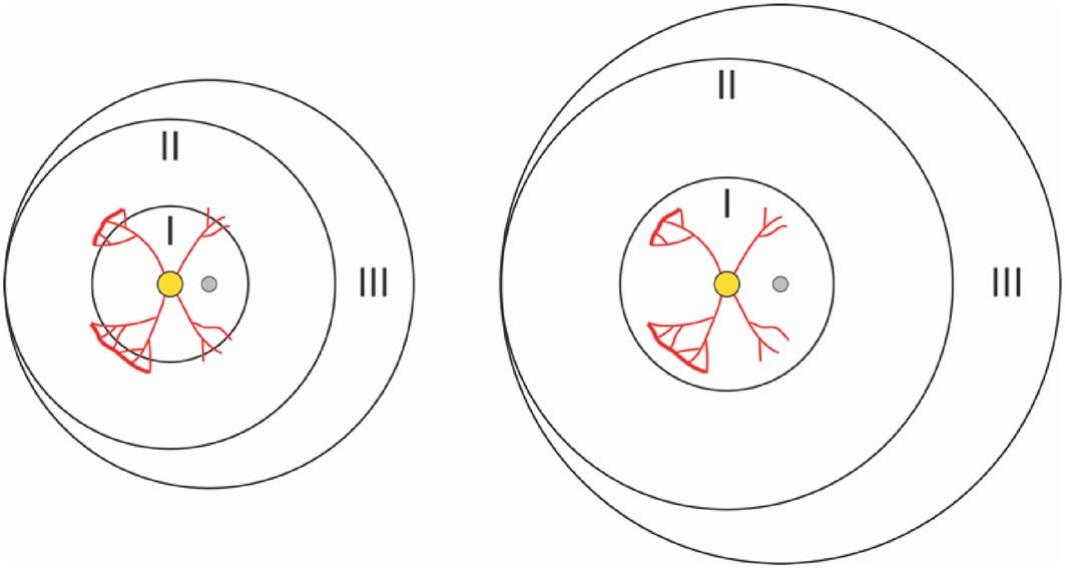
Neonatal eye size may affect zone designations. Schematic illustrating the hypothesis that the same absolute area of ROP involvement could be designated as Zone II in one eye but as Zone I in a larger eye. Zones are indicated by Roman numerals, optic disc in yellow, and macula in gray.

A similar hypothesis that arises from our model concerns Zone II, the region adjacent and extending outward from Zone I^12,13^. Because the threshold to intervene is lower in Zone I compared to Zone II ROP^37^, Zone II disease is consistently treated at later PMAs. This includes eyes with ROP in posterior Zone II, which typically undergo treatment around PMA 36 weeks versus 34 weeks for Zone I^38–43^. Since AL increases with PMA and zone areas should increase with AL, it is possible that the physical distance from disease to macula in early posterior Zone II cases may actually be less than in older babies with Zone I ROP (Fig. 5). If true, this would suggest that untreated Zone II ROP in smaller eyes or at early PMAs might be at higher risk than presently appreciated, a notion which again could be tested by a prospective study.

More broadly, it is worth considering the potential advantages of accounting for retinal area during ROP screening. Within the pediatric retina and ROP communities, it is widely thought that regions of retinal avascularity lead to increased levels of growth factors such as vascular endothelial growth factor (VEGF) that ultimately drive disease progression^44^. Current guidelines imply that eyes with the same proportion of avascular retina (e.g. vascularization of only the 30° radius circle corresponding to Zone I) carry similar risk of adverse outcomes regardless of eye size. However, different absolute areas of avascular retina may result in different amounts of growth factor elaboration, possibly influencing the course of ROP. Proportional risk in ROP is further predicated on accurately measuring the proportion of affected retina, analogous to placing a protractor inside the eye to measure the angle subtended. This accuracy is undermined when using the practical but subjective definition of Zone I from ICROP2, which depends on all ocular parameters affecting the light path in addition to the optics of the condensing lens. Finally, the proportion of the ocular interior covered by the retina is expected to decrease after PMA 30 weeks due to increasing size of the pars plana^45–47^. Consequently, the same 30° radius circle, even if perfectly measured, might not represent the same proportion of retina at different ages – an issue mitigated if absolute areas are used instead. While it is not feasible to directly measure retinal size in ROP patients, one strategy that might allow areas to be approximated is newborn imaging. This approach has its own inaccuracies such as correction of magnification^48^, but is capable of real-world implementation as demonstrated by its use in ROP telemedicine programs^35,49,50^.

Lastly, several limitations of the model used in this study should be mentioned. First, due to the challenges of directly measuring retinal size in neonates, we were unable to validate our area calculations against experimental measures. However, the estimates generated by our model are within reason. For example, at PMA 50 weeks, our model predicts the radius of Zone I to range from 6.40 to 7.89 mm, indicating a nerve-to-fovea distance of 3.20–3.95 mm. We find this range to be sensible for an eye of that age considering that the mean nerve-to-fovea distance in adults is around 4.76 mm^51^. In addition, a major assumption of our model is that ocular growth occurs evenly throughout the eye. While growth in reality is likely greatest in the retinal periphery^52^, newborn eyes with longer ALs do exhibit greater widths, heights, and surface areas^53^, suggesting growth in all directions. Furthermore, in children without myopia, eyes appear to enlarge globally in length, width, and height^54^.

In summary, we present a model to estimate the absolute area of retinal zones based on measurable properties of the neonatal eye. Using this model, we posit that accounting for retinal area or AL during ROP screening may be worth testing. Given the close relationship between zones and long-term vision in ROP, our study has implications for the management of this disease. A more precise understanding of zones in the context of retinal area or AL may enable the development of treatment strategies that also account for these variables, potentially improving outcomes.

## Supplementary Information

Supplementary Information.
