# Supplementary material for: Modeling absolute zone size in retinopathy of prematurity in relation to axial length
Source: Sci Rep. 2022 Mar 18;12:4717. doi: 10.1038/s41598-022-08680-5 (PMC8933429; doi:10.1038/s41598-022-08680-5)
Supplement: Supplementary file 1 — Supplementary Information. [file 41598_2022_8680_MOESM1_ESM.pdf]

Supplementary Equations

(1) Position of posterior nodal point

$$\text{Xnodal} = \frac{1.4000}{\frac{\text{Plens}}{1000} + \frac{1.3375}{\frac{1.3375}{\frac{\text{Pcornea}}{1000}} - \left(\text{ACD} + \frac{\text{LT}}{2}\right)}} + \text{ACD} + \frac{\text{LT}}{2}$$

$$- \sqrt{\left(\frac{1}{-\frac{\text{Pcornea}}{1000} + \frac{1.3375}{\frac{1.3375}{\left(-\frac{\text{Plens}}{1000}\right)} + \text{ACD} + \frac{\text{LT}}{2}}} + 100\right) \frac{\left(\frac{1.4000}{\frac{\text{Plens}}{1000} + \frac{1.3375}{\frac{1.3375}{\frac{\text{Pcornea}}{1000} - \frac{1}{100}} - \left(\text{ACD} + \frac{\text{LT}}{2}\right)}} + \text{ACD} + \frac{\text{LT}}{2} - \frac{1.4000}{\frac{\text{Plens}}{1000} + \frac{1.3375}{\frac{1.3375}{\frac{\text{Pcornea}}{1000} - \left(\text{ACD} + \frac{\text{LT}}{2}\right)}}} - \left(\text{ACD} + \frac{\text{LT}}{2}\right)\right)}{1.3375}}$$

(2) Area of Zone I

$$\text{A} = \pi * ((\text{AL} - \text{Xnodal}) * \tan 30^{o})^{2}$$

Supplementary Table 1. Equations used to calculate ocular parameters

Study	Parameter Equation
Gordon and Donzis, 1985	P_{cornea} = -0.0326(PMA^2) + 2.043(PMA) + 21.63
	P_{lens} (modified SRK) = 0.0844(PMA^2) - 7.3356(PMA) + 192.71
	AL = 0.0015(PMA^2) + 0.1193(PMA) + 9.6593
Inagaki, 1986	P_{cornea} = 0.0241(PMA^2) - 2.4781(PMA) + 107.66
Tucker et al., 1992	AL = 0.3(PMA) + 5.06
O'Brien and Clark, 1994	AL = -0.0002(PMA^2) + 0.1927(PMA) + 9.2571
Isenberg et al., 1995	ACD = 1.07+0.028(PMA)
	AL = 10.6 + 0.15(PMA)
Fledelius and Christensen, 1996	AL = -0.0086(PMA^2) + 0.8696(PMA) - 3.806
Cook et al., 2003	P_{cornea} = 0.0229(PMA^2) - 2.4688(PMA) + 111.11
	ACD = 0.0005(PMA^2) - 0.0026(PMA) + 1.5279
	P_{lens} (Hoffer Q) = 0.006(PMA^2) - 0.9275(PMA) + 69.969
	LT = -0.0008(PMA^2) + 0.0767(PMA) + 2.2593
	AL = -0.0012(PMA^2) + 0.2609(PMA) + 8.3308
Ozdemir et al., 2015	ACD = 1.9 + 0.029(PMA-28)
	LT = 3.3 + 0.039(PMA-25)
	AL = 15.05 + 0.152(PMA-28)
Kardaras et al., 2019	AL = 0.0051(PMA^2) - 0.1628(PMA) + 15.693
